# Effects of ligand coordination on Ag_8_SnS_6_ as a photoabsorber for thin film solar cells†

**DOI:** 10.1039/d5tc00397k

**Published:** 2025-03-12

**Authors:** Panagiotis Dallas, Vasileios K. Tzitzios, Lida Givalou, Polychronis Tsipas, Georgia Basina, Elias Sakellis, Nikos Boukos, Thomas Stergiopoulos

**Affiliations:** a Theoretical and Physical Chemistry Institute, National Hellenic Research Foundation, 48 Vassileos Constantinou Avenue, Athens 11635 Greece pdallas@eie.gr; b Institute of Nanoscience and Nanotechnology, NCSR Demokritos 15341 Athens Greece p.dallas@inn.demokritos.gr v.tzitzios@inn.demokritos.gr t.stergiopoulos@inn.demokritos.gr; c National Institute of Materials Physics, Atomistilor 405A Magurele Romania; d Department of Physics, National and Kapodistrian University of Athens Greece

## Abstract

Ag_8_SnS_6_ (ATS) nanoparticles, with a band gap of 1.35 eV, which is located exactly at the Schockley–Queisser optimal value for a single-junction solar cell, were utilized as a photoabsorber component in solid state photovoltaic devices. The as-made particles were capped with long aliphatic chains of oleic acid and oleylamine. After surface functionalization of the shorter and extremely basic formamidinium cations, an increase of the absorption coefficient throughout the visible spectrum range was observed. The ligand exchange led also to a slight increase of the band gap, by a value of 0.05 eV. XRD, XPS, UPS, diffuse reflectance, TEM and EDX characterization studies revealed the structure of the nanoparticles and finally proof-of-concept thin film solar cells were fabricated. A maximum photoconversion efficiency of 0.22% was achieved for the as-made particles.

## Abstract 2

Ag_8_SnS_6_ (ATS) nanoparticles, with a band gap of 1.35 eV which is located exactly at the Schockley–Queisser optimal value for a single-junction solar cell were utilized as a photoabsorber component in solid state photovoltaic devices.

## Graphical abstract

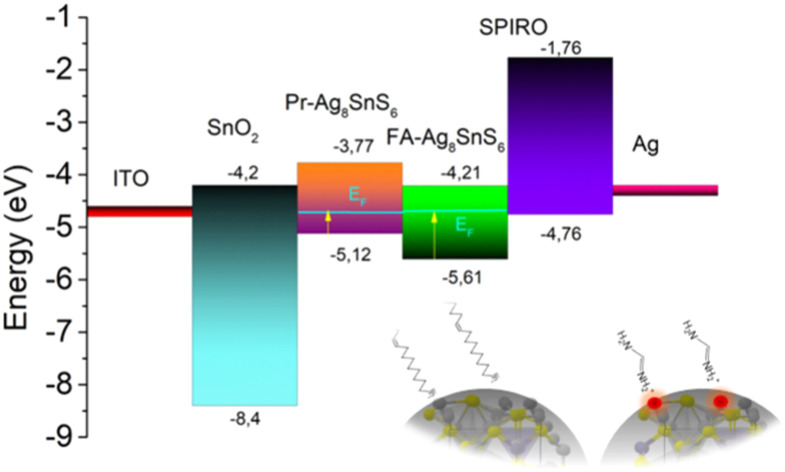

## 1. Introduction

Metal chalcogenides are an intriguing class of materials with applications in solar cells devices,^1^ light emitting diodes^2^ and photocatalysis.^3^ Chalcogenide nanocrystals possess inherent advantages, such as low cost of preparation, high absorption coefficient values that routinely reach the levels of 10^5^ M cm^−1^, surface functionality and a band gap that can be tuned at will.^4^ Specifically, in the field of solar cells, a series of ternary semiconducting chalcogenide nanoparticles such as AgBiS_2_^4^ and Cu_2_SnS_3_^5^ have been utilized as photoabsorbers, while the promising ABX_3_ (A = A  =  Ca, Ba; B  =  Zr, Hf, S = S, Se) have encountered difficulties forming high quality thin films for solid state photovoltaics.^6,7^

Inorganic perovskites such as BaZrS_3_ are known to demand high temperature solid state procedures to be synthesized. However, recent reports have demonstrated their solution-based synthesis towards colloidal nanoparticles, which is expected to provide new pathways for their applications in photovoltaics.^8^ On the other hand, AgBiS_2_ can be easily synthesized in solution with the ligands that are decorating its surface appearing to influence the photovoltaic performance.^9,10^ AgBiS_2_ recently has achieved a record efficiency of 10.2% when submicron grain-thin films through a vapor assisted solution process were applied,^11^ while cation-disorder-engineered colloidal nanocrystals reported a certified PCE value of 8.85%.^12,13^

An efficient solar absorber material for practical applications needs an energy gap, *E*_g_, near 1.34 eV to produce maximal output power.^14^ However, only a small number of binary semiconductors satisfies this requirement and limits the panel of materials suitable for solar absorbers in photovoltaics. However, an advantage of the ternary chalcogenides semiconductors is that the band gap can be tuned by varying the ratios of the constituent elements. To that end, the I–III/IV–VI (Cu- and Ag-based) ternary metal sulfides, have been extensively studied as candidates for applications in photovoltaics as well as light emitting diodes.^15^

Ag_8_SnS_6_ with the canfieldite crystal structure has an ideal *E*_g_ of 1.3–1.5 eV, and high absorption coefficients of *α* ∼ 10^4^ cm^−1^ in the visible range.^16^ Ag, Sn and S are non-toxic, relatively low-cost elements and environmentally friendly. With respect to the above, Ag_8_SnS_6_ nanocrystals have been utilized as counter electrodes in dye-sensitized solar cells,^17^ photocatalytic dye degradation,^18^ and photoelectrochemical salt-water splitting.^19^ Recently, Ag_8_SnS_6_ photovoltaic devices have been fabricated with an efficiency of 0.25%.^20^ Zhu *et al.* employed spiro-OMeTAD as hole transport layer and mesoporous TiO_2_ as electron transport layer to fabricate the thin film solar cells.

Here, we report a simple, straightforward, solution-based preparation of Ag_8_SnS_6_ nanocrystals and investigation of their photovoltaic properties. We investigated ATS nanoparticles capped with two different ligands. Specifically, we compared nanoparticles functionalized with the bulky, long, aliphatic chains of oleic acid and oleylamine with nanoparticles capped with the short formamidinium (FA^+^) cation. In both cases, photovoltaic devices were fabricated and a strong increase in the absorption coefficient was demonstrated after functionalization with FA^+^.

## 2. Experimental section

### 2.1. Synthesis of Ag_8_SnS_6_ nanoparticles

The canfieldite Ag_8_SnS_6_ nanoparticles were synthesized following a slightly modified, previously reported, methodology based on the utilization of elemental sulfur-amine solutions which has been applied for the synthesis of monometallic,^21^ as well as binary metal sulfides colloidal particles.^22^ Briefly, stoichiometric amount of Ag^+^ and Sn^4+^ in the form of AgNO_3_ (2 mmol), and SnI_4_ (0.25 mmol), were dissolved in well-degassed mixture of oleylamine–oleic acid, 18/1 v/v ratio, at 100 °C. The mixture remained under a continuous flow nitrogen blanket followed by the injection of elemental sulfur-oleyl amine solution, with 10% excess in sulfur (1.65 mgram-atom of sulfur in 3 ml oleylamine). Then, the temperature was raised up to 220 °C and remained at this temperature for 1 h. Finally, the solution was cooled to room temperature and the formed nanoparticles were precipitated by the addition of ethanol and separated by centrifugation. The process was repeated several times to ensure the removal of any reaction byproducts and unbonded amine and carboxylic acid molecules. The sample is denoted as Pr–Ag_8_SnS_6_.

### 2.2. Surface functionalization with formamidinium cations

In a typical experiment for the surface functionalization with shorter amine ligands, 100 mg of formamidinium acetate were dissolved in 4 ml of methanol. 1 ml of a stock Ag_8_SnS_6_ solution in toluene was added to 3 ml of chloroform. The two solutions were mixed under constant stirring for 3 hours. The nanocrystals were precipitated by centrifugation and washed twice with methanol to remove any unreacted organic molecules. The sample is denoted as FA–Ag_8_SnS_6_.

### 2.3. Fabrication of solar cells devices

All solar cell fabrication steps were performed in air. Indium tin oxide (ITO) covered glass substrates were cleaned by ultrasonication in an aqueous solution of soap, acetone and isopropanol for 20 min each. This was followed by 20 min UV-ozone treatment. SnO_2_ electron transport layer was then spin coated from an Alfa Aesar SnO_2_ colloid solution (1 : 5.6 v/v in H_2_O) at a spin coating speed of 2.000 rpm for 30 s and was further annealed at 180 °C for 15 min. 15 layers of Ag_8_SnS_6_ were deposited from 2 mg ml^−1^ toluene solution *via* the layer-by-layer method. For each Ag_8_SnS_6_ layer, Ag_8_SnS_6_ solution was spin coated onto SnO_2_/ITO substrates (2.000 rpm for 30 s). Then, 3-mercaptopropionic acid (3-MPA)/methanol (1% v/v) solution was applied to the film for 45 s, followed by two rinse–spin steps with methanol and once with toluene. The films were transferred into the glovebox for 10 min annealing at 115 °C and then stored in dry air overnight before spin coating the hole transport materials (HTM). Spiro-MeOTAD (36.6 mg ml^−1^ chlorobenzene stirring at 80 °C for 45 min), 14.5 μl *tert*-butyl pyridine (TBP), 9.5 μl LiTFSI solution in acetonitrile, was spin coated at 4.000 rpm for 15 s and stored in dry air overnight. Finally, 120 nm Ag were thermally evaporated on the HTL. The devices were stored in the dark in dry air and *J*–*V* measurements were recorded the day after the HTM deposition.

## 3. Characterization techniques

Fourier Transform Infrared (FTIR) spectra for the solid samples were measured on a Thermo Nicolet iS50 instrument in attenuated total reflection mode from 400 cm^−1^ to 4000 cm^−1^. X-ray diffraction (XRD) analysis was performed in the 2*θ* range of 2–80° with a Smart Lab Rigaku diffractometer (Cu Kα radiation; *λ* = 1.5418 Å). A Thermo Fisher Scientific FEI Talos F200i field-emission (scanning) transmission electron microscope (operating at 200 kV), was used for TEM and STEM imaging. It is equipped with a windowless energy-dispersive spectroscopy microanalyzer (EDX, 6T/100 Bruker, Hamburg, Germany). The dispersions of the pristine and composite materials were deposited on copper grids for the TEM analysis. UV-visible absorbance spectra were carried out on an Analytic Jena Specord 210 plus spectrophotometer with quartz cuvettes. X-ray photoelectron spectroscopy (XPS) measurements were carried out to analyze the chemical state and composition of the Ag_8_SnS_6_ films. The XPS data were collected with a PHOIBOS 100 (SPECS) hemispherical analyzer using Mg Kα X-ray source with photon energy 1253.64 eV. Voigt functions were used for the fitting analysis after standard Shirley background subtraction. Work function (Wf) and valence band maximum (VBM) values were extracted by Ultraviolet photoelectron spectroscopy (UPS) measurements using a Helium excitation source with He I radiation at 21.22 eV. The *J*–*V* curves were recorded with an Ossila solar cell *I*–*V* test system while the devices were illuminated by an AAA LED Solar Simulator, emitting AM 1.5 G light of 1000 W m^−2^ (Wavelabs Sinus-70).

## 4. Results and discussion

**Fig. 1:**
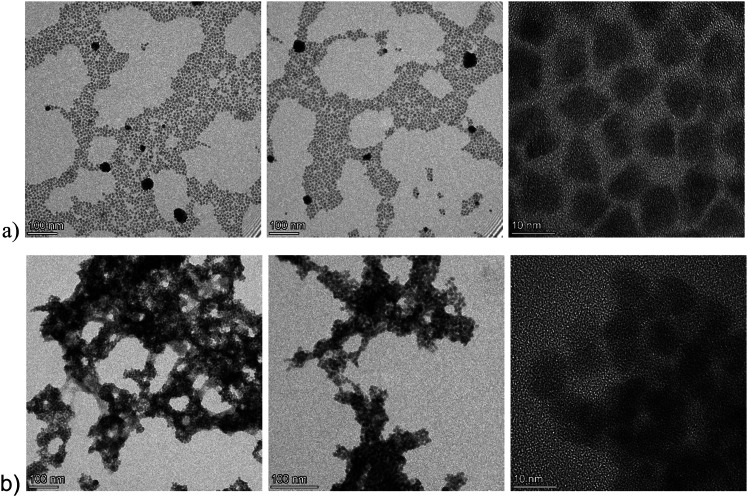
Representative TEM bright-field images of the pristine, Pr–Ag_8_SnS_6_, particles (a) and the functionalized FA–Ag_8_SnS_6_ (b). The particles appearing with high contrast are silver-rich phases that are formed under the influence of the electron beam.

Ag_8_SnS_6_ is a narrow, direct, bandgap semiconductor which has been widely used as light absorbers in fields of photovoltaic power generation and photocatalytic degradation of organic pollutants.^23^ In this work, the synthesis took place through high temperature decomposition of metal salts in the presence of strongly coordinating ligands such as oleic acid and oleylamine. The size and shape of the synthesized nanoparticles was revealed by TEM images that can be seen in Fig. 1a. An irregular shape for Ag_8_SnS_6_ nanoparticles is quite common in literature. For example, Yang *et al.*^24^ observed the formation of Ag_8_SnS_6_ polyhedra through a solvothermal synthesis utilizing tin(ii) bromide (SnBr_2_), silver nitrate (AgNO_3_) and thiourea (CS(NH_2_)_2_) as precursors, while Wang *et al.* synthesized triangular particles with silver acetate (C_2_H_3_AgO_2_), tin chloride dihydrate (SnCl_2_·2H_2_O) and thiourea in an oleylamine solution.^25^ On the other hand, spherical particles with a diameter and size distribution, depending on the reaction temperature, were synthesized through the decomposition of diethyldithiocarbamate complexes of tin and silver.^26^ In our case, a variety of sizes was identified, with most particles having a rectangular shape. As expected, after functionalization with the shorter formamidinium ligand, the particles are aggregated as can be seen in Fig. 1b.

**Fig. 2:**
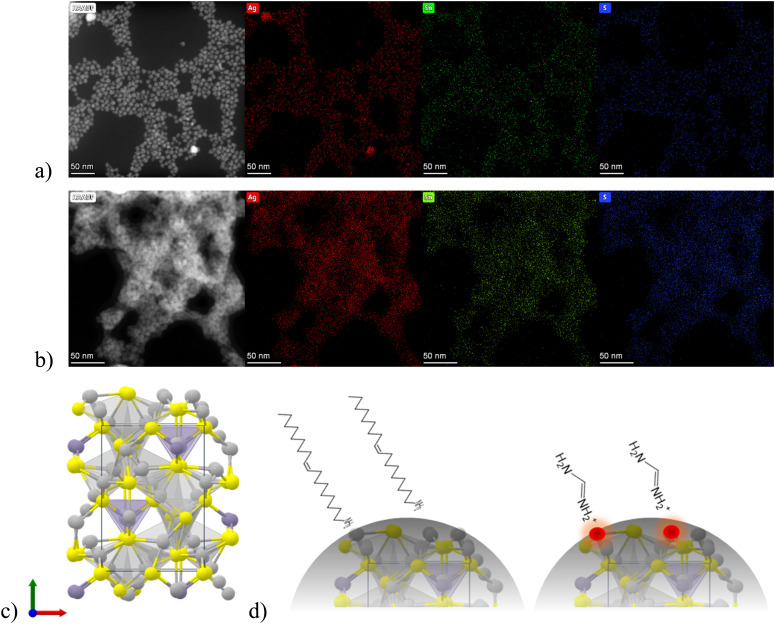
(a) and (b) STEM high-angle annular dark-field (HAADF) images and EDX mapping in two different areas. Ag; Sn; S are seen as red, blue and green areas respectively (c) atomic structure diagram of the Ag_8_SnS_6_ crystal. Ag: silver; Sn: purple; S: yellow (d) schematic representation of the coordination between an organic amine and the surface silver of ATS. The oleic acid molecules are omitted for clarity.

Furthermore, EDX mapping identified the presence and the distribution of the Ag, S and Sn elements in the crystallites of both Pr–Ag_8_SnS_6_ and FA–Ag_8_SnS_6_ (Fig. 2a and b respectively). The quantification of the three elements provided the following stoichiometry for the pristine and the formamidinium modified crystals: Ag_5.88_SnS_4.42_ and Ag_4.13_SnS_3.9_ respectively. This implies that the mobile and loosely coordinated silver cations are partially replaced by the organic cation, forming a hybrid material. Argyrodites comprise a large family of compounds with a general chemical formula of A_8_BX_6_ (A = Cu, Ag; B = Si, Ge, Sn; and X = S, Se, and Te). The materials, belonging to this family of chalcogenides, are well known as superionic semiconductors.^27,28^ In their crystal structure, the A^+^ cations are loosely bound compared to the strong Sn–S covalent bonds. In the work by Heep *et al.*, the bonding environment in Ag_8_SnS_6_ was pictured through electron localization function (ELF) and crystal orbital Hamilton population (COHP).^29^ This revealed that the charge clouds of Sn–S bonds are delocalized, and the Sn is covalently bonded to the S atoms. Furthermore, the Ag atoms are discrete, and no electron was found to be localized near the Ag atoms. In this work, we report a case where the surface is coated either with a carboxylate group and a long amine or the same carboxylate ligand and a short amine. In Fig. 2c the atomic structure diagram of the ATS crystal is presented, alongside the proposed coordination modes in the two aforementioned cases (Fig. 2d).

**Fig. 3:**
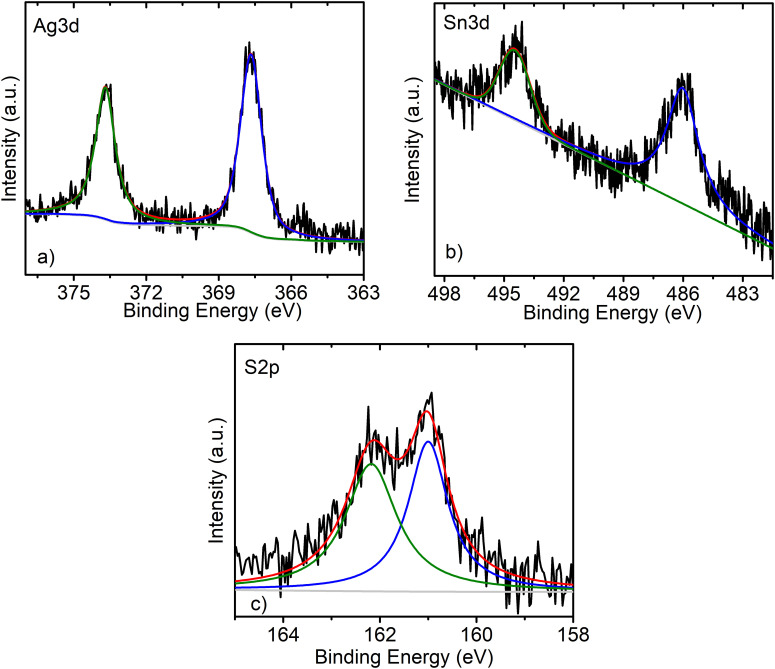
XPS spectra of the (a) Ag 3d, (b) Sn 3d and (c) S 2p peaks recorded from the sample Pr–Ag_8_SnS_6_.

A more detailed structural characterization was made viable with XPS surface analysis. In Fig. 3a we present the XPS spectra corresponding to the Ag 3d, Sn 3d and S 2p peaks of the Pr–Ag_8_SnS_6_ sample.^24,30^ The Ag 3d spectrum demonstrates two clearly distinguished components related to the Ag 3d_5/2_ and Ag 3d_3/2_ core energy levels due to the spin–orbit coupling. Table 1 summarizes and compares the values obtained in our work with those reported in two representative papers on the XPS analysis of Ag_8_SnS_6_. The binding energy values of Ag 3d are shifted to lower binding energy values compared to those reported in literature.^24,30^ This shift is assigned to the increased electron density of the metal cation after functionalization with electron donating amine ligands.^31^ The analysis reveals that the Sn^2+^ peak typically located at 485.7 eV is absent, signaling that all tin is in the Sn^4+^ state. The S 2p spectrum demonstrates binding energies similar to Ag–S bonding similar to Ag_2_S crystals, while the absence of components belonging to S–O bonds in the range of 165–171 eV excludes the formation of any oxysulfide species.^32^

**Table 1:** Comparison of the BE (eV) values with two representative previously published papers

	Ag (3d_3/2_ & 3d_5/2_)	Sn (3d_3/2_ & 3d_5/2_)	S (2p_1/2_ & 2p_3/2_)
Pr–Ag_8_SnS_6_	373.72 & 367.67	495.26 & 486.04	162.2 & 161
Ref. 25	373.9 & 367.95	495.95 & 487.15	162.8 & 161.9
Ref. 19	374.3 & 368.3	495.4 & 486.9	162.9 & 161.8

**Fig. 4:**
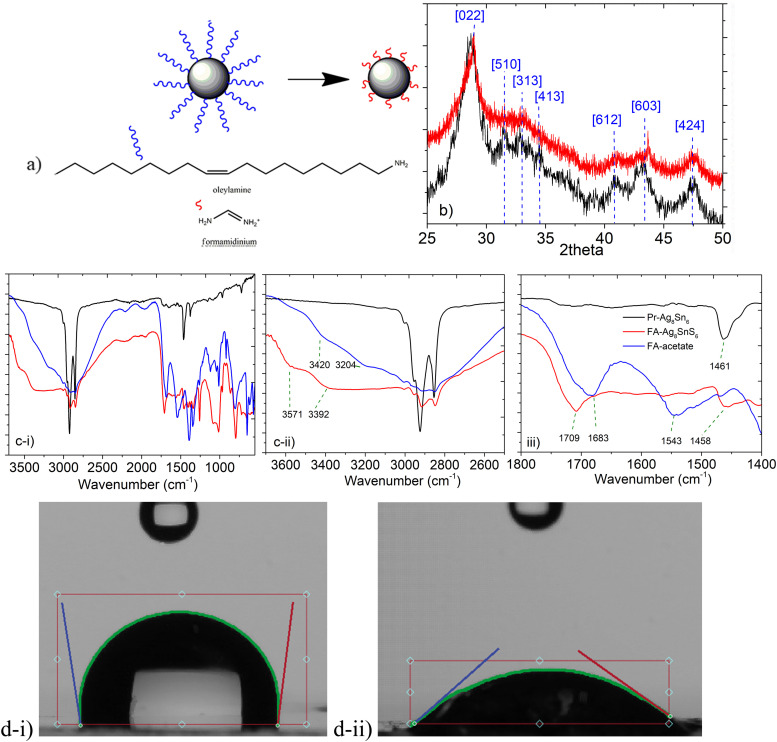
(a) Schematic representation of the exchange of oleylamine with the formamidinium cations (b) XRD patterns of the Pr–Ag_8_SnS_6_ and FA–Ag_8_SnS_6_ samples. (c) FTIR spectra in three different areas (d) contact angle measurements for Pr–Ag_8_SnS_6_ (i) and FA–Ag_8_SnS_6_ (ii).

The XRD pattern of the Pr–Ag_8_SnS_6_ nanoparticles reveals the typical diffraction expected from its canfieldite orthorhombic phase. Ag_8_SnS_6_ crystallizes in the orthorhombic *Pna*2_1_ space group with a crystal structure involving eight inequivalent Ag^+^ sites. There is a spread of Ag–S bond distances ranging from 2.44 to 2.95 Å. After the ligand exchange with FA^+^ (Fig. 4a), we observed that the patterns of Pr–Ag_8_SnS_6_ and FA–Ag_8_SnS_6_, are identical, with the only exception of a slight shift towards smaller *d*-spacings for the [603] diffraction peak (Fig. 4b). After the ligand exchange, the FTIR spectra (Fig. 4c) demonstrates the stronger presence of amine groups, and a reduced intensity of the aliphatic chains, an observation based on the reduced intensity of the –C–H groups, while both the –N–H bending and the –N–H stretching, that relate to primary amines, appear to shift towards higher energies. This is outlined in Table 2. Furthermore, the imine C=N mode of the formamidinium cation appears to shift at higher energies after surface functionalization. Specifically, the broad peak at 1683 cm^−1^ of the pristine ligand, is observed at 1703 cm^−1^ in the case of the FA–Ag_8_SnS_6_ sample. Contact angle measurements with water, demonstrated that after the cation exchange, the particles are becoming significantly less hydrophobic, something that proves the successful substitution of the aliphatic surfactants with the more polar formamidium cations. The images can be seen in Fig. 4d and reveal a decrease of the contact angle from 97.89° for the Pr–Ag_8_SnS_6_ to 38.64° for the FA–Ag_8_SnS_6_.

**Table 2:** The values corresponding to the vibrational modes of N–H stretching, N–H bending, C–H bending and C–N of formamidinium acetate, Pr–Ag_8_SnS_6_ and FA–Ag_8_SnS_6_ are presented for clarity

Sample	N–H stretching (cm^−1^)	N–H bending (cm^−1^)	C–H bending (cm^−1^)	C–N (cm^−1^)
Pr–Ag_8_SnS_6_	—	—	1461	—
FA–Ag_8_SnS_6_	3393/3571	1709	1458	1123
FA–acetate	3204/3420	1683	—	1079/1012

**Fig. 5:**
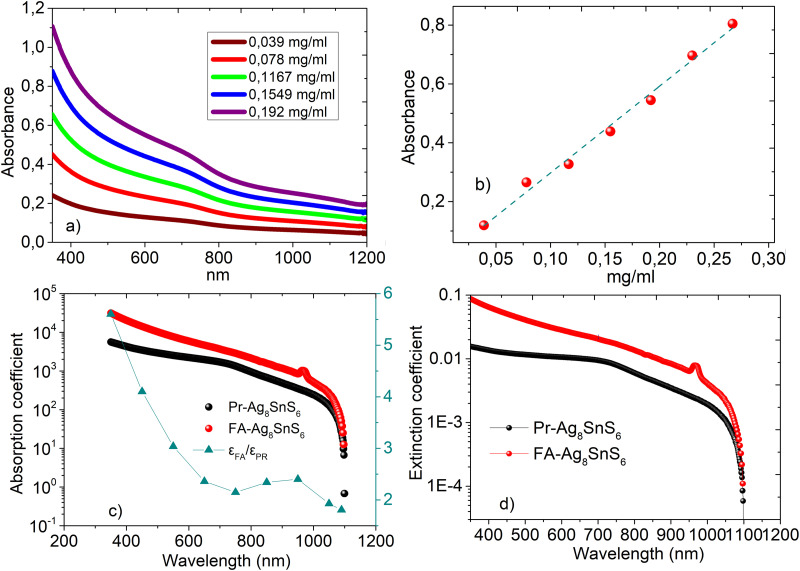
(a) UV-vis spectra and the corresponding calibration curves (b). Absorption coefficient (*ε* in M^−1^ cm^−1^) (c) and extinction coefficient (*k* in M^−1^) (d) values in the range 380–1200 nm for the Pr–Ag_8_SnS_6_ and FA–Ag_8_SnS_6_. The ratio between *ε*_FA_/*ε*_Pr_ can be seen in Fig. 5c.

The UV-visible absorption spectra of the nanoparticles can be seen in Fig. 5a. The shoulder located at 710 nm is similar to the one reported in the work of Liu *et al.*^27^ Calibration curves were constructed based on the absorbance at *λ* = 450 nm and can be seen in Fig. 5b. For the construction of the calibration curves, we subtracted the value at *λ* = 1200 nm from the absorbance intensity values to avoid the contribution of scattering. Nanomaterials exhibit a very high surface to volume ratio and thus the percentage of atoms on the surface is substantial and the presence of defects and the difference in the dielectric environment greatly affect their electronic, optical, thermal and chemical properties.^33^ For example, halide perovskites functionalized with sodium dodecylsulfonate (SDS), exhibited significantly increased fluorescence intensity and an external quantum yield of 8.4%.^34^ When used in light emitting diodes, the SDS molecules have stronger absorption energy on CsPbI_3_ perovskites compared to oleic acid and thus, they suppress the defect formation due to ligand loss during the nanoparticles purification process. Furthermore, plasmonic chalcogenides such as CuFeS_2_ demonstrated a red shift of the surface plasmon resonance after electrostatic interaction with covalent organic frameworks and sodium dodecylsulfonate.^22^ Interestingly, after ligand exchange of the bulky and aliphatic oleylamine with the small and electron dense formamidinium cation, we observed an increase on the absorption and extinction coefficient values, see Fig. 5c and d. This is in accordance with a previous work by Kroupa *et al.* who studied the optical absorption enhancement in PbS NPs upon ligand exchange from oleate to a series of cinnamate capping agents. Through experimental work and *ab initio* simulations, the authors concluded that the optical absorption enhancement was due to a coupling between the NPs energy levels of the ligand considering the ligand-nanoparticles dyad as a distinct chemical system.^35^ For clarity, we present the ratio between the absorption coefficient of the two samples (*ε*_FA_/*ε*_Pr_) in the right axis of Fig. 5c. We observed an absorption coefficient 2.4 times higher in the case of the modified sample close to the band gap (*λ* = 950 nm). In the same logic with the fabrication of the calibration curves, the absorption values at low energies corresponding to scattering were subtracted throughout the spectral range. The extinction coefficient *κ*, was calculated *via* the equation 
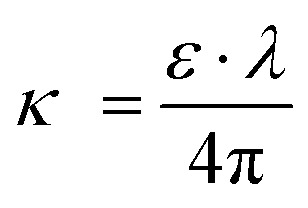
; *ε* is the molar absorption coefficient, *λ* is the wavelength in cm. Based on the FTIR observations that were earlier discussed, we assign this increase to the strong binding of the electron donating formamidinium ligand which, in addition, does not possess long aliphatic chains like the oleylamine. Similar phenomena have been also observed in BaTiO_3_–*x*Bi_2_O_3_ ceramics, where the absorption increased with increasing the percentage of bismuth.^36^

**Fig. 6:**
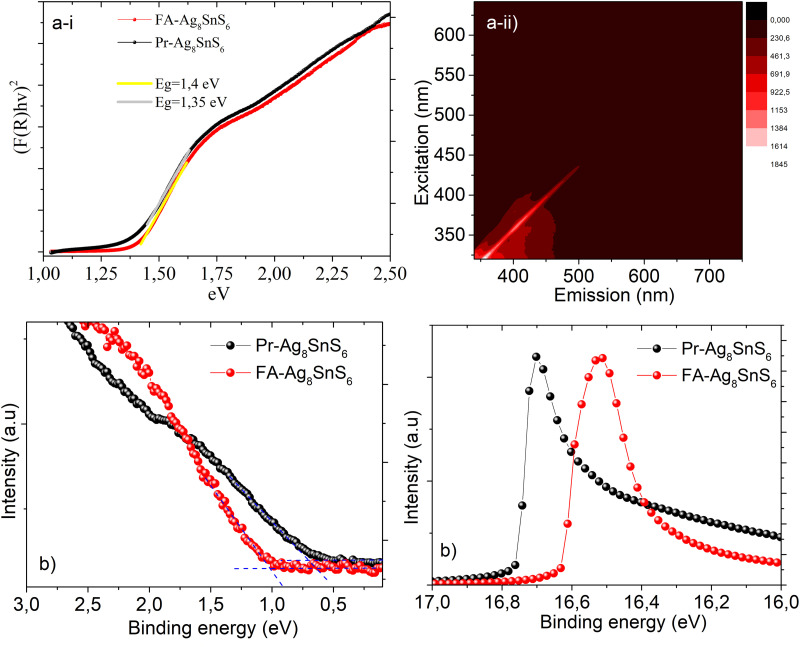
(a) (i) Tauc plots of Pr–Ag_8_SnS_6_ and FA–Ag_8_SnS_6_ and the calculated band gap. (ii) Excitation dependent photoluminescence maps. (b) UPS spectra of Pr–Ag_8_SnS_6_ and FA–Ag_8_SnS_6_ for the calculation of Fermi level (left) and valence band maximum (right).

The argyrodite Ag_8_SnS_6_ is considered an ideal band gap semiconductor for photovoltaic applications. We proceeded to identify the energy levels and the Fermi level position of both the Pr–Ag_8_SnS_6_ and the FA–Ag_8_SnS_6_ samples through diffuse reflectance spectra and Ultraviolet Photoelectron Spectroscopy. The Tauc plots obtained from the diffuse reflectance values can be seen in Fig. 6a. After the exchange of organic ligands with the formamidinium cations, we observe a slight increase of the band gap from 1.35 eV to 1.4 eV, with a negligible Urbach tail. The lack of the exponential Urbach tail indicates the absence of amorphous, largely disordered areas.^37^ Finally, the excitation dependent photoluminescence mapping for the Pr–A_8_SnS_6_ can be seen Fig. 6aii. No detectable photoluminescence was observed throughout the ultraviolet and the visible light range. The same behavior was observed for the FA–Ag_8_SnS_6_. Preliminary measurements up to *λ* = 1600 nm again did not reveal any photoluminescence at room temperature.

Ultraviolet Photoelectron Spectroscopy is a powerful tool for the calculation of the valence band, the work function and the Fermi level of a material. In order to record the spectrum, the samples were drop-casted on conductive substrates of FTO. In Fig. 6b the UPS spectra of the two samples are presented. The work function (Wf) is calculated from subtracting the secondary energy cutoff from the He I radiation of 21.22 eV.^38^ In the case of the Pr–Ag_8_SnS_6_, the Fermi level is located 0.66 eV higher than the valence band, while the work function was calculated at 4.46 eV. Taking into consideration the band structure of the Pr–Ag_8_SnS_6_ nanocrystals, we observe that the Fermi level lies in between the valence and conduction band. Interestingly, the coordination with the smaller and highly basic formamidinium alongside the partial removal of silver cations, is leading to a re-alignment of the energy bands, with the Fermi level now lying 1.02 eV higher than the valence band, transforming the surface of the nanoparticles into a highly n-type semiconductor.

**Fig. 7:**
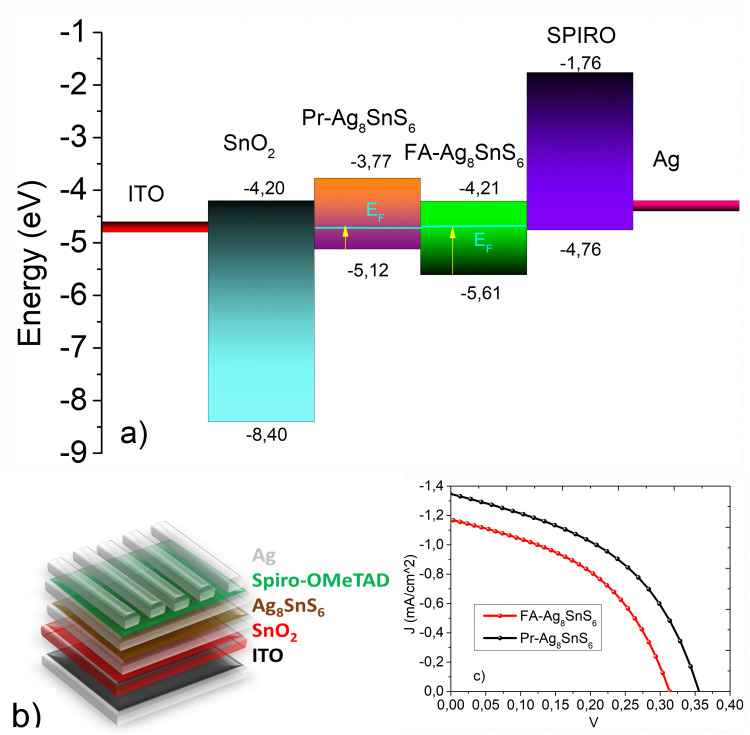
(a) The energy levels alignment. The band structure of both nanoparticles is included in the graph (b) schematic representation of solar cell devices (c) *J*–*V* curves.

Finally, we proceeded with the fabrication of thin films photovoltaic devices. SnO_2_ was employed as an electron transport layer on ITO-covered glass substrates. According to the UV-visible reflectance spectra and the UPS analysis, the band structure of the nanoparticles was calculated and in Fig. 7a, the correlation between the energy levels of all components of the device is depicted. The exact structure of the photovoltaic device can be seen in Fig. 7b. The long aliphatic chains in the case of Pr–Ag_8_SnS_6_ were removed after exchange with the short ligand 3-mercaptopropionic acid. Spiro-MeOTAD was employed as the hole transport layer. In the previous work by Zhu *et al.*^20^ regarding the use of ATS as a photoabsorber in solid-state solar cells, titanium dioxide (TiO_2_) was used as an electron transport layer and Spiro-OMeTAD as the hole transport layer, the former being deposited on fluorinated tin oxide (FTO) glass. The band alignment between the nanocrystals, the ETL SnO_2_ and the HTL Spiro-OMeTAD^39^ clearly demonstrates that the position of the valence band maximum and the conduction band minimum of the Pr–Ag_8_SnS_6_, enables the electron and hole transfer and hence a charge separation and transport towards the electrodes can be achieved. This appears to be hindered in the FA modified sample. The *J*–*V* curves for both samples can be seen in Fig. 7c, with the corresponding *V*_oc_, *J*_sc_, PCE and FF values are gathered in Table 3. We attribute the slightly higher PCE and FF values of the Pr–Ag_8_SnS_6_ to its ability to form more uniform films after spin-coating, due to the long aliphatic chains present on its surface. These long insulating chains do not significantly affect the conductance of the material, since they are exchanged with the shorter 3-mercaptopropionic acid after the thin film formation. Furthermore, the UPS studies revealed that the energy levels of the formamidinium modified nanoparticles moved towards more negative values, lying exactly in the level of the conduction band of the electron transport layer, SnO_2_. However, we argue that the slightly lower current and fill factor, extracted from the FA-based compound, is due to the high n-type character of the surface of the film; in a n–i–p solar cell configuration we have adopted here, a p-type surface would be better for hole extraction towards the HTM. The Pr–Ag_8_SnS_6_ photovoltaic performance was tested 2 months after the fabrication and presented an efficiency of 0.17% (Fig. S1, ESI†). Despite a severe FF loss, the initial PCE remains almost unchanged. This is showing promise for the real-life applications of the absorber.

**Table 3:** A summary of the *V*_oc_, *J*_sc_, PCE and FF values

Sample (solvent)	*J*_SC_ (mA cm^−2^)	*V*_OC_ (V)	PCE (%)	FF (%)
Pr–Ag_8_SnS_6_	1.35	0.36	0.22	44.5
FA–Ag_8_SnS_6_	1.17	0.31	0.16	43.3

## 5. Conclusions

Canfieldite colloidal nanoparticles with different types of ligands were found to exhibit an optimal band gap in the range of 1.35 to 1.4 eV with no detectable photoluminescence in the visible light range. The surface functionalization of Ag_8_SnS_6_ nanoparticles with electron donating formamidinium cations increased the absorption coefficient throughout the visible spectrum and transferred the energy bands of the nanoparticles towards more negative values. Furthermore, the surface functionalization altered the solubility behavior of the particles rendering them dispersible in more polar solvents such as DMF. Both formamidinium and oleic acid/oleylamine capped ATS nanoparticles can be utilized as photoabsorbers in solar cells with the bulky aliphatic chains enabling a slightly increased fill factor and photoconversion efficiency.

## Data availability

Data for this article are either included in the manuscript or are available upon request by the corresponding authors.

## Conflicts of interest

There are no conflicts to declare.

## Supplementary Material

TC-013-D5TC00397K-s001
