# Supplementary material for: Effects of ligand coordination on Ag8SnS6 as a photoabsorber for thin film solar cells
Source: J Mater Chem C Mater. 2025 Mar 12;13(16):7996–8005. doi: 10.1039/d5tc00397k (PMC11915202; doi:10.1039/d5tc00397k)
Supplement: TC-013-D5TC00397K-s001 [file TC-013-D5TC00397K-s001.pdf]

Supplementary Information (SI) for Journal of Materials Chemistry C.
This journal is © The Royal Society of Chemistry 2025

Effects of Ligand Coordination on Ag_8SnS_6 as a Photoabsorber for Thin Film Solar Cells

Panagiotis Dallas*[ab], Vasileios K. Tzitzios*[b], Lida Givalou[b], Polychronis Tsipas[bc], Georgia Basina[ab], Elias Sakellis[bd], Nikos Boukos[b] and Thomas Stergiopoulos*[b]

[a] Theoretical and Physical Chemistry Institute, National Hellenic Research Foundation, Athens 11635, Greece

[b] Institute of Nanoscience and Nanotechnology, NCSR Demokritos, 15341, Athens, Greece

[c] National Institute of Materials Physics, Atomistilor 405A, Magurele, Romania

[d] Department of Physics, National and Kapodistrian University of Athens, Greece

- pdallas@eie.gr; p.dallas@inn.demokritos.gr; v.tzitzios@inn.demokritos.gr; t.stergiopoulos@inn.demokritos.gr

Highlights: Ag_8SnS_6; photovoltaics; canfieldite colloids; chalcogenides; semiconductors, ligand exchange

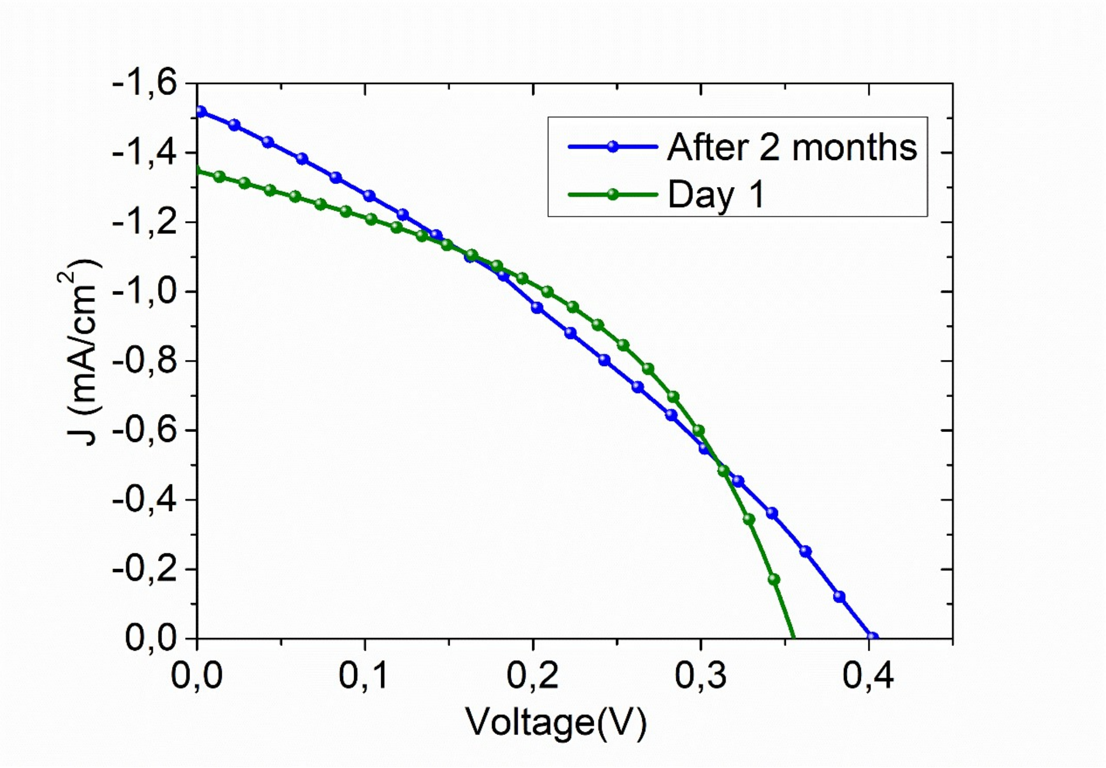

Figure S1. J-V curve recorded after two (2) months compared to the one recorded after one (1) day of solar cells fabrication. Sample: Pr-Ag_8SnS_6

	**PCE (%)**	**FF (%)**	**J_{sc} (mA/cm^2)**	**V_{oc}(V)**
**After 2 months**	0,17	29	1,52	0.40
**Day 1**	0,22	44,5	1,35	0.36

Table S1. PCE; Fill factor (FF), J_{sc} and V_{oc} values. Sample: Pr-Ag_8SnS_6
